# Long-Term Recovery Patterns and Limited Spillover of Large Predatory Fish in a Mediterranean MPA

**DOI:** 10.1371/journal.pone.0073922

**Published:** 2013-09-12

**Authors:** Antoni García-Rubies, Bernat Hereu, Mikel Zabala

**Affiliations:** 1 Centre d’Estudis Avançats de Blanes, (CEAB-CSIC), Blanes, Spain; 2 Departament d’Ecologia, Universitat de Barcelona, Barcelona, Spain; Technical University of Denmark, Denmark

## Abstract

Based on 19 y of visual census data from the Medes Islands MPA (NW Mediterranean), this study analyzes the carrying capacity (K) and population recovery time of six species of fish strongly affected by harvesting pressure along the Mediterranean coast. Three of these species (*Epinephelus marginatus, Diplodus cervinus* and *Dicentrachus labrax*) have practically reached carrying capacity in the Medes Islands MPA, while others are still approaching population stabilization (*Sciaena umbra*) or are still increasing in biomass (*Dentex dentex*). The one exception to these trends is *S. aurata*, which tended to decrease inside the MPA, probably due to fishing just outside its borders. These results confirm that fish populations may require decadal time scales to recover from exploitation, both in terms of total abundance (21 to 29 y to exceed 95% K) as well as total biomass (25 to 35 y), and that rates of recovery differ between species (13 to 31 y). The recovery and saturation observed within the no-take zone contrasts with results obtained in the partially protected buffer area and the peripheral area open for fishing, which show much lower biomass values. In general, the spillover from the MPA is very moderate, and its effects extend only to the partially protected area.

## Introduction

Marine Reserves have been proposed as an effective conservation tool [1]–[3] in the face of increasing degradation of marine ecosystems due to overfishing [4]–[7]. Although there is considerable evidence of the effectiveness of MPAs in rebuilding stocks (i.e. an increase species richness, density, size and biomass) (reviewed by [8]), other expectations such as total recovery of populations or the export of biomass, have proven much more difficult to demonstrate, with some existing controversy about the actual usefulness of reserves as fishery-management tools.

The export of biomass from the MPAs is based on two hypothesized processes. First, enhanced spawning stock biomass inside MPAs may result in an export of eggs and larvae, boosting recruitment into fishery stocks [9], [10], [11]. The export of larvae from MPAs has been very difficult to conclusively demonstrate [12], although there is some evidence of it for fish [13] and mollusks [14]–[16].

Second, MPAs may export adult fish to areas open to fishing, through a process called *spillover*. Spillover may occur either by density-independent movements such as *home range*, ontogenic movements and seasonal reproductive migrations [17], [18], [19], or by density-dependent movements once the habitat is saturated within the MPA [19]–[21]. If the mobility of the species is high and fishing pressure around the MPA is very strong, any effect of protection on the *rebuilding* of populations may be considerably reduced or undetectable [22], [23]. Even if the process of *rebuilding* is evident, *spillover*, caused by density-dependent processes, will occur only when the population within the MPA has reached carrying capacity [24], [25].

However, attaining carrying capacity is not merely important as a precursor to *spillover*. According to McClanahan et al. [25], identifying the rate of recovery of fish in MPAs is fundamental, among other processes, for designing MPA networks, fisheries management, research on interactions, and for providing managers a clear basis for evaluating the effectiveness of protection. The recovery rate is also a benchmark for assessing the status of populations outside MPAs and the time required for exploited populations to reach their full recovery.

No consensus exists about how long protected populations require to reach full recovery, which can vary from 1 and 3 y [26], 4 to 6 y [27], or much longer, reaching decades [28], [29]. According to Russ and Alcala [28], this disparity is possibly due to the fact that most studies are based on short-term research that was conducted soon after a protection was put into place, although some evidence exists for relatively rapid recoveries, which can be explained by environmental factors and species-specific biological features [30]. Thus some authors have emphasized that regular, well-designed and long-term monitoring studies in areas with a long history of effective protection are the surest way to properly document the time required for populations to reach their carrying capacity in MPAs [28].

In this study we monitored six highly targeted fish species inside and outside a MPA with a history of 25 y of strict protection with an aim to 1) document cases of population saturation within the MPA, 2) assess the carrying capacity for 6 high value target fish species, 3) estimate the time required to reach the carrying capacity for these six species and 4) verify the existence of *spillover* from the MPA to areas open to fishing.

## Materials and Methods

### Study Sites and Sampling Method

**Figure 1:**
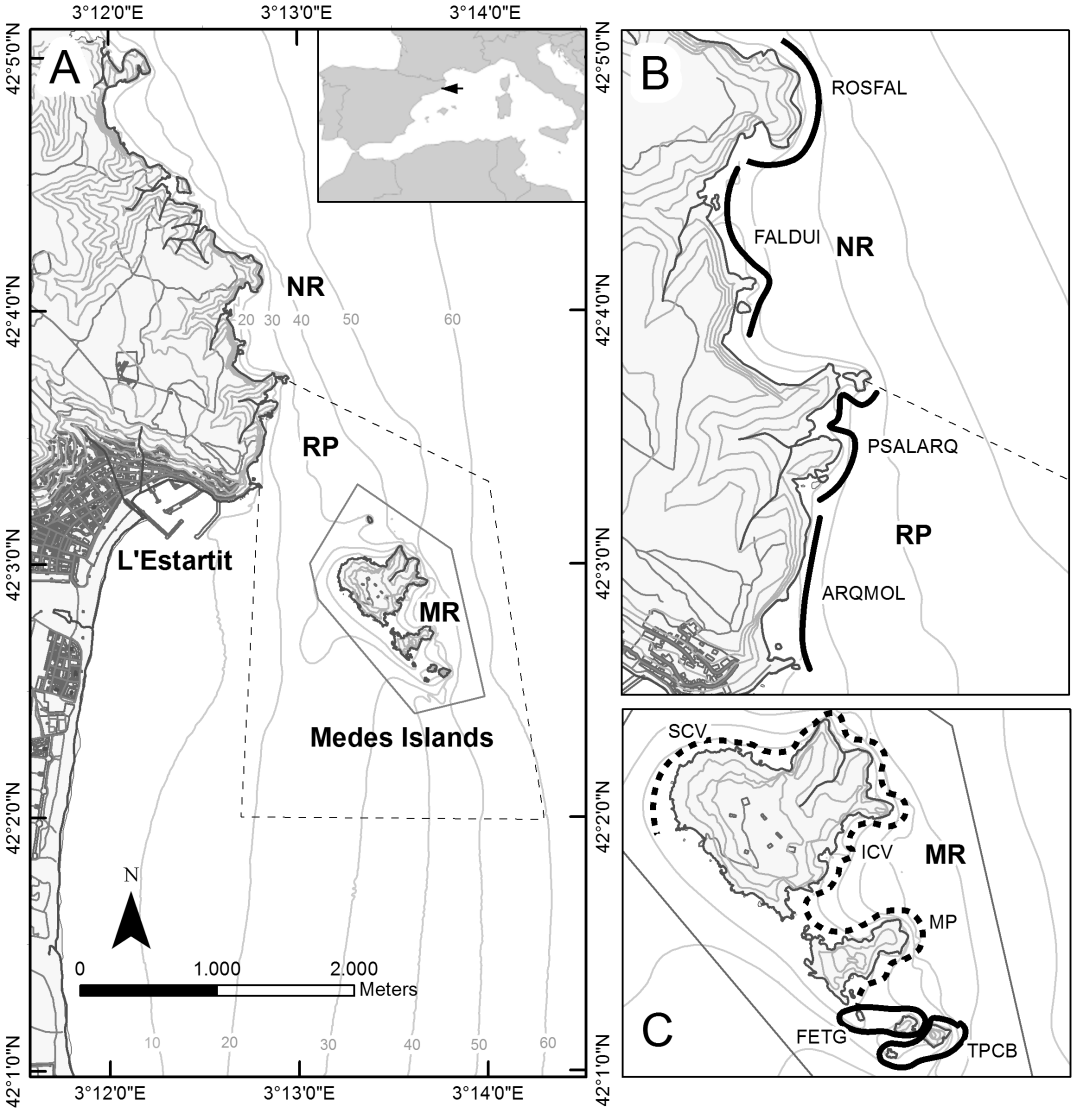
Study site. Medes Islands Marine Reserve. A) General view. Gray solid line represents the limits of the Marine Reserve (MR) where all fishing is prohibited, and gray dashed lines represents the limits of the Partially Protected Reserve (PR) where fishing is regulated, delimiting the non protected area (NP). B) Transects in the Non-Protected Area (NP) and in the Partially Protected Area (PR). C) Transects inside the Marine Reserve (MR). Black solid lines represent replicated transects, and black dashed line represent non-replicated transects.

The Medes Islands are a small archipelago formed by two islands (Meda Gran and Meda Petita) and a series of minor islets (Carall Bernat, Tascons, Ferranelles and the Medellot), located approximately 1,5 km from the coastal town of l’Estartit on the Costa Brava in NE Catalonia (Figure 1). The Medes Islands marine reserve (MR, a fully protected marine area) was created in 1983. In 1990, the perimeter of the reserve was expanded, and a Partially Protected Reserve *buffer zone* (PR) was established in the section closest to the islands of the neighboring coast of Montgrí (Figure 1), with the aim of facilitating possible *spillover* from the marine reserve. Within the Marine Reserve, 95 ha are fully protected, and all forms of mining/harvesting activity and anchoring is completely prohibited, while permitting other activities such as marine tourism, swimming and scuba diving.

The Medes Islands have been the focus of a number of scientific papers, some of which [31] have been instrumental in the establishment of the marine reserve. The effect of protection on its littoral fish community was demonstrated shortly after the reserve was implemented [32], [33]. The protection and recovery of the demographic structure of the grouper population (*Epinephelus marginatus*) in the area were crucial for documenting the reproduction of this species for the first time in the Mediterranean [34], [35], and determining its principle environmental drivers [36]. The effect of protection on the fish fauna has also been the focus of several research projects designed to compare Mediterranean MPAs [37], [38], [39].

In 1992 a monitoring of the populations of highly targeted fish species began within the protected area of the Medes Islands MPA (MR). In 1999, the monitoring was extended to include the partially protected area (PR), and a portion of unprotected coast (Figure 1). The monitoring was conducted continuously from 1992 to 2009, with gaps in some areas due to logistic constraints.

The six species chosen for this study (*Epinephelus marginatus, Dentex dentex*, *Dicentrarchus labrax, Diplodus cervinus, Sciaena umbra* and *Sparus aurata*) were selected based on the results of two earlier studies [32], [33], which showed that these species were very sensitive to the effect of protection, and that their populations were considerably higher inside the MPA than outside it, where they were rarely encountered in censuses. All the selected species are long-lived, can reach a considerable size and are prized for their culinary qualities, making them especially vulnerable to fishing [40].

The sampling was based on 8 to 10 transects of 50 m in length per site, located at depths between 10 m to 20 m. The observer swum each transect along the bottom, recording individual species found within a 10 m belt, 5 m on either side of the transect, thus covering an area of 500 m^2^. Sizes were estimated to within 2 cm for every observed individual up to 50 cm, and within 5 cm for larger individuals.

Two sites were established in each of the three levels of protection (Marine Reserve, Partial Reserve and No Reserve). In the Medes Islands Marine Reserve, counts were conducted around the smaller islands (FETG and TPCB; Figure 1b); on the coast of Montgrí, two zones were established in the Partial Reserve (PSALARQ and ARQMOL), located approximately 1 km from the Medes Islands and two more in the Non Protected area open to fishing (ROSFAL and FALDUI) (Figure 1c). These counts were repeated four times between July and August each year. In the protected area of the Medes Islands Marine Reserve (MR), in addition to these two areas, three additional sites were established (MP, ICV and SCV) at which a single annual count was performed, following the protocol detailed above (Figure 1b). The study was restricted to rocky bottoms (blocks and walls) with similar environmental characteristics between the different levels of protection [37].

This study was conducted as part of the Medes Islands Marine Reserve monitoring program; thus, all necessary research permits were obtained from authorities responsible for the Marine Reserve and the adjacent unprotected coast (Departament de Medi Natural, Generalitat de Catalunya). Field work was based on visual census techniques that required no animal handling. In addition, we made no animal or plant collections for this study.

### Data Analysis

We used species richness (S), species abundance and total biomass per census as synthetic descriptors for each site. At the species level, we only considered biomass values, which were calculated from the length-weight relationship for each species [41] and expressed in units of fresh weight per unit area (g × m^−2^). Results with abundance values were completely redundant with those obtained from biomass and hence are not presented here.

We analyzed temporal changes in these descriptors in the three levels of protection, checking the fit of the total average values with various functions using the Levenberg-Marquardt least-squares algorithm in the statistical package STATISTICA 6.0. In the Medes Islands reserve, in addition to temporally replicated areas (the average of 4 annual inventories), we also included the non-replicated areas in the analysis. The functions tested were as follows:

The linear function *y* = a+bt, which assumes a constant rate of increase or decrease (b) at time (t) from an initial value (a).
The exponential function (*y* = a.*e* ^(rt)^), which assumes a growing increase or decrease as a function of time (t), where r is the rate of change and *e* is Euler's number.
The limited growth model of von Bertalanffy, y = K(1 − *e* ^(−r(t − t0))^), which has rapid initial growth that reduces with time as it approaches the value of K, which is the theoretical maximum value *y* that can be reached, i.e., the carrying capacity of the system, where t is the elapsed time and t_0_ is the theoretical time where *y* = 0.
The limited growth logistic curve model y = K/(1+(K/*y_0_* − 1)*e* ^(−rt)^ ), similar to the previous model as *y* reaches a maximum theoretical value K from an initial value (y_0_) of the dependent variable but with a similar start as that of an exponential function, which gives rise to a characteristic sigmoidal curve.

For all the models, the time elapsed (in years) was considered from the establishment of the Medes Islands MPA (1983). The function chosen in each case was that which, being significant (according to an F test, adjusted for degrees of freedom), best explained the observed variance (R^2^). For those indicators that fit best to asymptotic functions, the model was projected over time to estimate when the population would reach 95% and 99% of the estimated value of K.

To analyze the effect of different levels of protection on the degree of rebuilding and the possible spillover out of the MPA, the mean number of species, abundance and mean total biomass of the six species were compared using permutational multivariate analysis of variance (PERMANOVA [42]) in areas temporarily replicated at each level of protection and year, based on the matrix of Euclidean distances between samples. The model was mixed with two fixed orthogonal factors: protection (P), with three levels (total protection: MR; partial protection: PR, no protection: NR), year (T: 1999–2009) and a 'zone' factor, which was random and nested in protection (Z(P)). Because, in this design, the number of permutations possible for the protection factor was limited (90), the Monte Carlo method was applied to increase the number of permutations. This analysis was conducted using the statistical package PRIMER-E Ltd. [43]. To maintain a fully balanced design (n = 4 per site per year) in the two sites for each protection level (MR, PR and NR) and to avoid an excessive number of zeros in the inventories, values obtained in the 50×10 m counts made each year were averaged for each site. The data from 1998 (for which only PR information was available) and 2007 (for which only partial information was available from some sites) were excluded from the analysis.

## Results

### Temporal Change in Populations in the Three Levels of Protection

**Figure 2:**
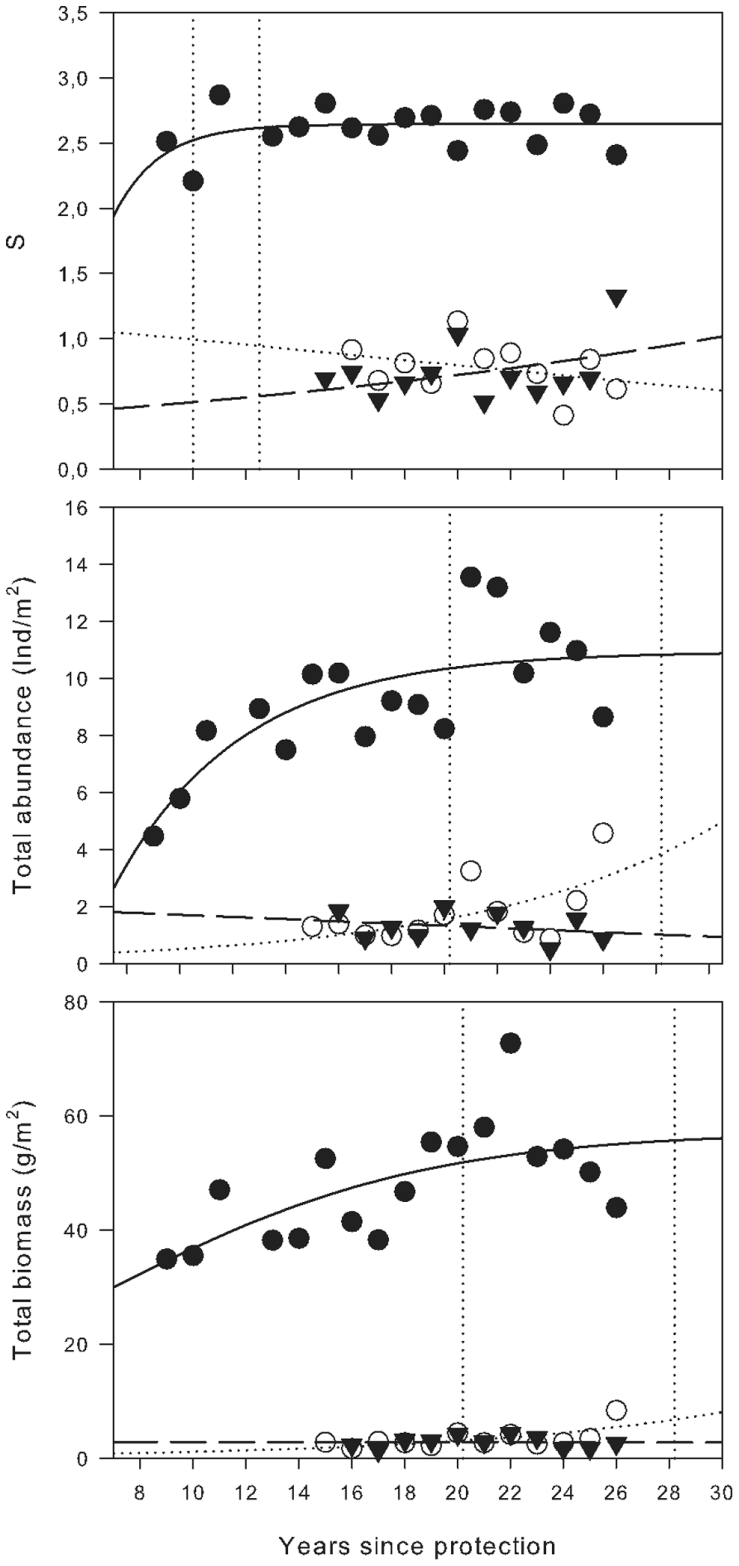
Temporal patterns of number of fish species vulnerable to fishing. Species richness (S, mean number), total abundance (Ind/m^2^) and total biomass (g/m^2^) of fish species vulnerable to fishing vs. duration of reserve protection for Marine Reserve (MR, solid circles), Partially Protected Reserve (PR, open circles) and Non Reserve (NR, solid triangles). Lines represent the best-fit logistic growth fitted to number of species of vulnerable fish on MR (solid line), PR (dashed line) and NR (dashed line) (functions described on table 1).

Species richness, total abundance and biomass showed a similar temporal pattern in MR areas, fitting well to asymptotic models (Table 1, see Appendix S1 for the results of whole models). Thus, the average species richness remained very stable in MR after an initial increase, allowing for a significant fit to the von Bertalanffy function, although the explained variance was low (R^2^ = 0.12). Annual average values of species richness settled around the value of K (2.65 species 500 m^−2^), which was reached in a relatively short time (T_95%K_ = 10 y; T_99%K_ = 13 y). Species richness tended to increase in PR, and there was a slight linear decrease over time in NR, with notable annual variations (Figure 2a).

**Table 1:** Explained variation (R^2^), K values (in asymptotic models), and significance of the models fitted for the different descriptors used in this study (T_95%_ and T_99%_ time in years to reach 95% and 99% of K in asymptotic models).

Descriptor	Model	R^2^	K	p	T_95%K_	T_99%K_
S	V. Bertalanffy	0,12	2,65	<0,001	10	13
Total abundance	V. Bertalanffy	0,58	10,94	<0,001	21	29
Total biomass	Logistic	0,42	57,26	<0,001	25	35
*E. marginatus* (abundance)	Logistic	0,68	3,13	<0,001	21	24
*E. marginatus* (biomass)	Logistic	0,38	37,28	<0,001	18	25
*D. dentex* (abundance)	Exponential	0,61		<0,001		
*D. dentex* (biomass)	Exponential	0,78		<0,001		
*D. cervinus* (abundance)	V. Bertalanffy	0,28	3,08	<0,001	12	14
*D. cervinus* (biomass)	Logistic	0,28	1,31	<0,001	13	16
*D. labrax* (abundance)	Logistic	0,24	2,08	<0,001	15	17
*D. labrax* (biomass)	Logistic	0,28	3,40	<0,001	20	25
*S. umbra* (abundance)	Logistic	0,62	3,96	<0,001	65	82
*S. umbra* (biomass)	Logistic	0,47	5,16	<0,001	31	51
*S. aurata* (abundance)	Exponential	0,43		<0,001		
*S. aurata* (biomass)	Exponential	0,18		<0,001		

The same temporal pattern was observed in the mean abundance of fish (Table 1). In MR, abundance fit the von Bertalanffy function (R^2^ = 0.58) with a K value of 10.9 ind. 500 m^−2^, 95%, which was reached in 21 y and 99%, reached at 29 y. The mean abundance also tended to increase in PR. This was mainly due to increases in *S. umbra* and *D. dentex* in 2009, and another sudden increase in *D. labrax* individuals in 2004. A slight declining trend in overall average density was observed in NR (Figure 2b).

The mean biomass in MR fit the logistic function well (R^2^ = 0.42), with an estimated carrying capacity of 50.3 g m^−2^, 95% of which surpassed in the 25 y of protection and, according to the model, will exceed 99% after 35 y (Table 1). Biomass remained virtually unchanged and with very low values for the entire monitoring period, both in NR (eg 2,6 g m^−2^, in 2009) and PR (eg 3,4 g m^−2^ in 2008), except for a rise in PR in 2009 (8,4 g m^−2^) (Figure 2c).

**Figure 3:**
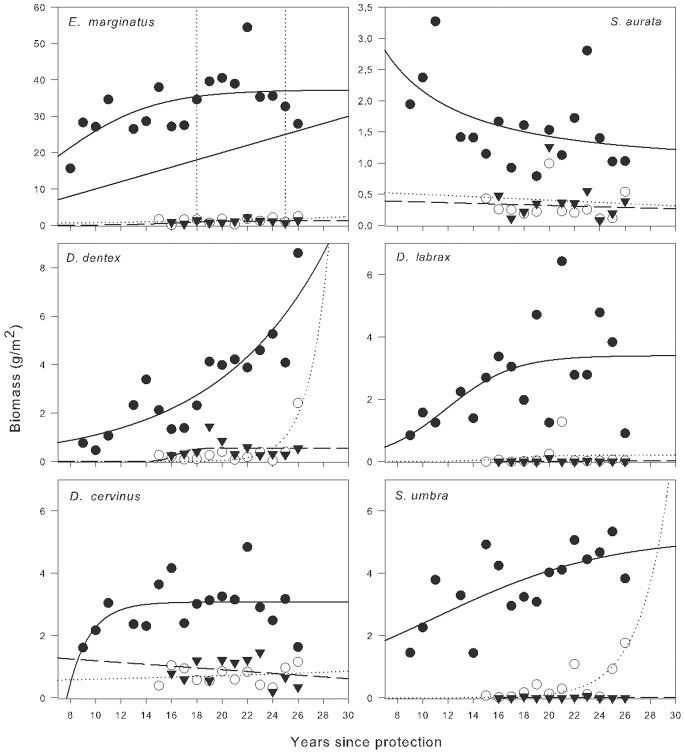
Temporal patterns of biomass of individual fish species vulnerable to fishing. Biomass of each of fish species vulnerable to fishing monitored vs. duration of reserve protection for Marine Reserve (MR, solid circles), Partially Protected Reserve (PR, open circles) and Non Reserve (NR, solid triangles). Lines represent the best-fit logistic growth fitted to number of species of vulnerable fish on MR (solid line), PR (dashed line) and NR (dashed line) (functions described on table 1).

Considered separately, temporal trends of mean biomass for most species in MR also conformed to asymptotic functions, with two exceptions: *Dentex dentex*, which tracked an exponential growth (R^2^ = 0.78), strongly influenced by a significant increase in 2009 (Table 1), and *Sparus aurata*, which fit a negative exponential function (R^2^ = 0.18), its biomass paradoxically declining inside the protected area (Figure 3).

The average biomass of *E. marginatus* appeared to fit well to the logistic model (R^2^ = 0.38), with a high K value (37.3 g m^−2^) achieved over the long term (T_95%K = _18 y; T_99%K = _24 y). Other species that showed similar trends were *D. cervinus*, which tracked a logistic curve (R^2^ = 0.28) that trended to a much smaller carrying capacity (K = 3,1. g m^−2^) and was achieved more rapidly (T_95%K = _13 y; T_99%K = _16 y), *S. umbra*, with a trend in biomass that fit to a logistic model (R^2^ = 0.47) and made an estimated full recovery after a much longer time than other species (K = 5.2 g m^−2^; T_95%K = _31 y; T_99%K = _51 y), and *D. labrax*, with a biomass change that tracked a logistic model (R^2^ = 0.28), despite marked interannual variations, with a relatively high carrying capacity (K = 3.4 g m^−2^) achieved in the medium term (T_95%K = _15 y; T_99%K = _17 y) (Figure 3).

In PR, some positive trends were observed in the change in biomass, with marked variation, for *E. marginatus* and *D. cervinus* and especially for *D. dentex* and *S. umbra*. In contrast, in NR, only the biomass of *E. marginatus* showed a slight upward trend, while other species remained at very low values with no clearly defined trends. Both PR and NR values were significantly lower than those observed in MR (Figure 3, Table 1).

### Differences Between Protection Levels and Spillover

There were marked differences between fish populations inside MR compared with PR or NR. On average, the number of species in PR and NR was between 28 and 30% of that observed in MR; the mean abundance in PR only reached 18% of that found in MR, and was even lower in NR (12% the abundance in MR). The total average biomass observed in PR and NR was between 6.7 and 5.4%, respectively, of that observed in MR.

PERMANOVA comparisons indicate that protection had a major influence on average species richness, total abundance and total biomass results (Table 2). For all these synthetic measures, the differences between the levels of protection were significant despite high variability between zones within in each level. Pair-wise tests confirm that these values were significantly higher in MR than in NR and PR (MR >PR = NR). For the most part, these differences remained relatively constant, without an interaction between protection and time. The one exception was mean species richness, where there was an interaction between time and protection due to an increase in the mean number of species in PR in 2004, which was significantly higher than NR (MR >PR>NR).

**Table 2:** Results of PERMANOVAs assessing the effects of Protection (total, partial and no protection, df = 2), Year (df = 9), Zone (random, nested within Protection, df = 7), Protection x Year (df = 18) and Zone x Year (df = 27) for 15 metrics of vulnerable fish (error df = 180).

	Protection		Year		Zone		Protection x Year		Zone x Year		Error
	MS	F	MS	F	MS	F	MS	F	MS	F	MS
Species number (S)	143,41	178,42***	0,283	1,616	0,804	6,296***	0,383	2,19*	0,175	1,371	0,128
Total Biomass	231,29	39,672**	0,63	1,923	5,83	26,384***	0,453	1,382	0,328	1,484	0,221
Total Abundance	92,97	41,671*	0,414	1,7168	2,231	18,927***	0,371	1,5367	0,241	2,0469**	0,118
*E. marginatus* Abundance	46,577	49,607**	0,116	3,404**	0,939	32,381***	0,028	0,83	0,034	1,178	0,029
*E. marginatus* Bionmass	263,57	39,761**	0,616	1,961	6,629	31,967***	0,319	1,017	0,314	1,515	0,207
*D. dentex* Abundance	6,573	3,639	0,28	1,243	1,806	17,787***	0,26	1,152	0,225	2,218**	0,102
*D. dentex* Biomass	46,097	6,153	1,251	2,359*	7,492	34,107***	0,637	1,202	0,53	2,415***	0,22
*D. cervinus* Abundance	9,925	21,527*	0,177	2,001	0,461	6,782**	0,171	1,935	0,088	1,299	0,068
*D. cervinus* Biomass	24,625	33,499*	0,334	1,859	0,735	5,237**	0,329	1,829	0,18	1,282	0,14
*D. labrax* Abundance	44,161	40,88**	0,912	5,089**	1,08	8,74***	0,616	3,436***	0,179	1,45	0,124
*D. labrax* Biomass	70,345	166,49***	0,749	4,135**	0,423	2,619	0,592	3,27**	0,181	1,123	0,161
*S. umbra* abundance	47,675	50,112**	0,273	2,315*	0,951	10,988***	0,272	2,309*	0,118	1,36	0,087
*S. unmbra* Biomass	76,908	69,833**	0,315	1,939	1,101	9,38***	0,35	2,156*	0,162	1,382	0,117
*S. aurata* Abundance	1,324	11,771*	0,168	4,171**	0,112	2,924*	0,075	1,868	0,04	1,048	0,038
*S. aurata* Biomass	7,897	69,905**	0,389	3,805**	0,113	1,437	0,147	1,44	0,102	1,3	0,079

* : 0.05>p>0.01.

** : 0.01>p>0.001.

*** : p<0.001.

These trends between protection regimes were confirmed at the individual species level for the 6 selected species. The effect of protection was significant for the biomass values of all species except *Dentex dentex*, which was marginally insignificant (p = 0.09), due to large variations in biomass that occurred in MR and a notable increase in 2009 in PR (Table 2). Biomass differences between protection regimes were maintained over time for *E. marginatus, D. cervinus* and *S. aurata*. For these species, the values observed in MR significantly exceeded PR and NR values (MR >PR = NR). *Dicentrarchus labrax* and *S. umbra* were exceptions. In the first case, there was a significant interaction between protection and time due to an increased average biomass in PR in 2004, which offset differences between MR and PR. This pulsed episodic rise was due to an escape of individuals from a fish farm relatively close by, and the increase was detected in both PR and MR but not in NR (Figure 3). In the case of *S. umbra*, the interaction between time and the level of protection was due to an increase of the species in PR in 2002 that was repeated in the last two years (2008 and 2009) and coincided with a marked decline of the species in MR (Figure 3) in 2009.

## Discussion

### Carrying Capacity

After 25 y of protection in the MR, the mean species richness, abundance and total biomass of the six studied species appear to have practically reached carrying capacity (Table 1). The average number of species per census was the fastest parameter to reach saturation (10–13 y), showing that the frequency of occurrence of these species in censuses recovers long before density and size. These results agree with McClanahan et al. [25], who estimated the recovery of the average number of species in several MPAs of Kenya at 10 y.

It is difficult to compare the biomass values obtained in this study, which focused on only 6 species, with those of other studies that normally account for the biomass of the entire fish community. Still, the biomass values we observed here are similar to comparable studies in the Medes Islands [37], [39] and match among the highest proposed estimates for Mediterranean MPAs located in coastal rocky habitats [44], [39].

For three species (*E. marginatus, D. labrax* and *D. cervinus*), biomass appears to have made a complete recovery within the MR, and while *S. umbra* is still increasing, its biomass is showing the first signs of stabilizing over the long term (T95%K = 31 y; T99% = 51 y). Were it not for an unusual spike in the biomass of *D. dentex* in 2009 inside the MR the trends in this species closely approximated a logistic growth; the high values of 2009 forced the fit to an exponential model instead.

The carrying capacities of these populations (K values) varied considerably between species. The grouper, *E. marginatus* had the highest value (K = 37.1±3.6 g m^−2^), which is an order of magnitude above the other studied species, such as *S. umbra* (K = 5.6±3.1 g m^−2^), *D. labrax* (K = 3.4±0.66 g m^−2^) and *D. cervinus* (K = 3.1±0.24 g m^−2^), suggesting that biomass values at the time of full recovery is highly dependent on the size and age that a species can reach [26]. The populations of *E. marginatus* have shown a dramatic recovery in many other MPAs in the NW Mediterranean where surveillance has been effective, such as Port-Cros [45], Cabrera [46] and Cabo de Palos [38], but the average biomass values we regularly recorded in the MR are extraordinary even when compared with these hotspots.

**Figure 4:**
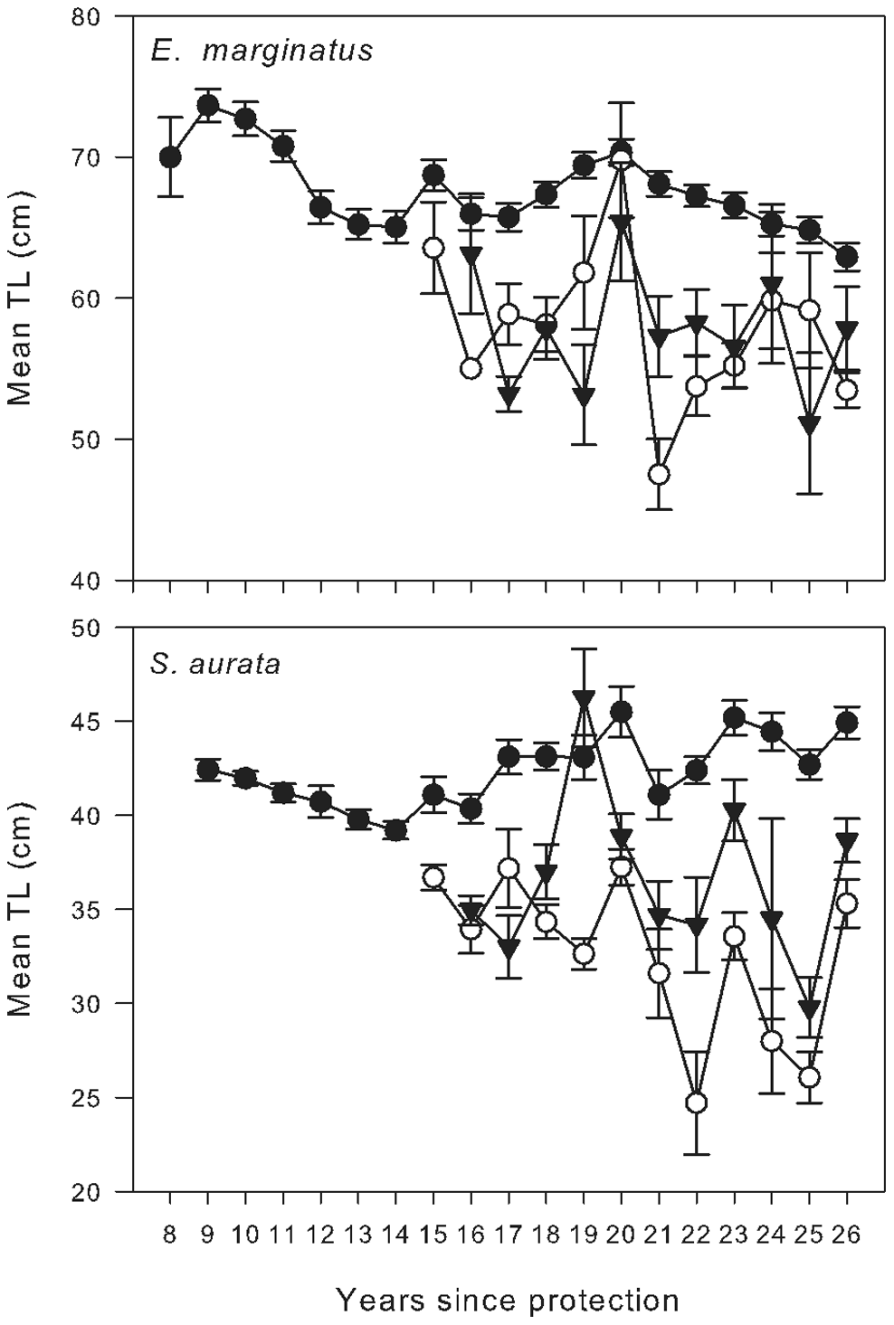
Mean size (± SE) by year of dusky grouper (*E. marginatus*) and sea bream (*S. aurata*) observed in the Marine Reserve (MR, solid cycles), the Partially Protected Reserve (PR, open circles), and the Non Reserve (NR, solid triangles).

The mechanisms underlying this successful rebuilding of the MIMPA is difficult to resolve with our long-term data. For *E. marginatus*, the biomass in MR at the start of monitoring (1991) was already much higher than the monitored areas outside (Figure 3) but continued to increase in subsequent years. This remarkable increase in overall biomass could have been mainly due to the growth of existing individuals, although our annual surveys showed that mean abundance increased similarly to biomass, while the average size even decreased somewhat from the initial years (Figure 4a). This suggests that the initial increase in biomass was mainly due to the appearance of new individuals in the Medes populations.

This is puzzling, since in all these years of censuses, not a single recruit (*young of the year* or YOY) of *E. marginatus* has ever been observed in MR, and individuals less than 40 cm (at least three years old) are rare. Young individuals are also scarce in the outlying areas within a radius of at least 20 Km, safely ruling out the possibility that this recovery is driven by self-recruitment even at regional scales. In the case of two other species (*D. cervinus* and *S. umbra*), juvenile records are so rare that it is problematic to explain how such dense populations of adults could build up inside the MPA. The most plausible answer is that many individuals are recruited from outside the MPAs, possibly from quite distant areas. This would involve a long-distance migration or wandering during certain phases of the life cycle, which as yet have not been described in the literature for these species. The lack of a regular annual recruitment may be a critical factor explaining why the process of reaching carrying capacity is so slow.

Although the population recovery time was highly species specific, it is clear that all species required a protracted period of protection to reach their carrying capacity (Table 1), ranging from 13 to 31 y to exceed 95% of K. Similar values were defined by Russ and Alcalá [28] or some of the estimates by McClanahan *et al*. [25], which ranged between 17 and 37 y. Our results underscore how critical long-term protection is for the populations of these heavily fished species in the Mediterranean.

These results contrast with studies that claim far shorter recoveries within MPA boundaries [21], [47]. This is perhaps due to a confusion of full recovery with the first signs of improvement in protected populations, which can usually occur relatively quickly. However, in a comparable study in terms of methodology, geographical proximity and temporal consistency, Coll et al. [30] indicated that the time for total biomass to reach capacity in three MPAs in the Balearic Islands (NW Mediterranean) varied among the different areas of the three reserves studied between 6 and 8 y, considerably lower than our estimates. These differences may be due to both environmental and biological factors. In the Balearic MPAs, the sampling occurred at more shallow depths (from 5 to 10 m) because the bedrock rarely reached lower depths. The limited depth of bedrock in the Balearic reserves was a bottleneck for the presence of the most long-lived species like large specimens of *E. marginatus*, which tend to move deeper as they grow [48], and large shoals of *S. umbra*, which require substrates with high structural complexity that rarely occur at such shallow depths. Moreover, it is notable that Coll et al. [30] took into account all of the species targeted by fisheries, including many short-lived species, that reach their maximum size relatively quickly (e.g., *Diplodus sargus, D. vulgaris*, etc.) and that, consequently, recover their demographic structure much faster than the species discussed in this study.

Not all the species we tracked have reached carrying capacity yet, and the case of the sea bream (*Sparus aurata*) seems paradoxical because, after reaching elevated biomass values in the first 2 y of monitoring in MR (8 to 9 y after protection), this species suffered a sudden drop after which it showed a continuous decline (Figure 3). The biomass of this species in MR appears to converge with PR and NR areas, the only significant difference between protection regimes being the larger size of individuals in MR. According to Babcock et al. [47], these declines, rare in a protected environment, may be due to three factors: initial increases in abundance may have been driven by factors other than protection (e.g. abnormally high recruitment), unconsidered side effects of protection (for instance an increase in predators) or an intensification of fishing around the reserves.

In the case of *S. aurata* the most plausible explanation of this reduction is fishing, to which it is subject when it aggregates to spawn. One of these aggregations is situated very close to the MR and is well known by purse seine fishermen in the area. The aggregation can be easily located using modern *full-circle scanning sonar*, and it requires very little effort to fish out a large number of individuals in a single fishing event (Sacanell, pers. obs.). In this case, *S. aurata* represents an example of density-independent export of biomass (as defined by Abessamis and Russ [49]), and is an example of the limitations of MPAs when part of the population sporadically leaves its boundaries or when the area protected is smaller than the dispersion capacity of the species in question [47]. For this species in particular, it can be asserted that if fishing on reproductive aggregations of this species is unhalted, the differences between MR and the areas open to fishing will be diluted over time.

In summary, carrying capacity is highly contingent on the species, as well as other factors governing the abundance of fish populations in a given area (e.g., topography, bathymetry ranges, benthic community, hydrographic regime, etc. [30]). Determining these species-specific carrying capacities requires a well-established MPA, long-term studies and the assurance that protection conditions are strictly adhered to throughout the duration of the study. These conditions are rarely met [50], which perhaps explains why literature addressing these issues is rare.

It may be argued that the fact that a growth curve is smooth for a few consecutive years does not guarantee that a final asymptote exists nor does it assure that a state of saturation has been reached. While this may be true for any single species, when the growth curves of several populations of target species in a region show similar patterns of saturation, the trends looks much more robust to individual variations.

### Effects of Protection

The number of species observed per census, was three times higher in the MR than PR or NR, while overall abundance was 5 to 6 times higher and biomass 13 to 19 times higher inside MR. These results are similar than those found in the most successful MPAs [8], [39], and highlight the inefficiency of partially protected zones for the full recovery of fish fauna [28], [39].

All of the studied species have, to a greater or lesser degree, been favored by the protection of the Medes Islands. Assuming that the regional biomass maxima of each species have been achieved in the MR, we can estimate the status of the populations in the areas open to fishing [51]. According to Worm et al. [52], an exploited stock whose numbers do not reach 10% of the unexploited biomass (pristine biomass, according to McClanahan et al., [25]) can be considered to be practically in ecological collapse. On this scale, the populations of *D. labrax*, *E. marginatus* and *S. umbra* fall between the categories of seriously depleted and collapsed in areas open to fishing, further underscoring the effectiveness of MPAs for the protection and future viability of these species.

The vast differences in fish biomass between MR and the coast are mainly due to top-down processes (i.e. fishing/protection) but quite possibly also favored by bottom-up factors such as those derived from the insularity. Located more than 1.5 Km offshore, the Medes Islands are more exposed to currents, which can increase both production and the supply of food for planktophagus species, which are the staple diet of most piscivores. The mechanisms involved in this phenomenon are varied and related to upwelling, vertical mixing, internal waves, benthic nutrient regeneration, run-off and eddies (e.g. [53]), and some studies have also demonstrated the consequences of enhanced zooplankton and fish larval densities for the survival of these organisms and their importance for local fisheries [54], [55]. It is not mere coincidence that the MPAs in which higher biomass values of fish are observed in the Mediterranean are islands [38]. However, it is protection that allows for the full potential of these *hotspots* to be manifested for harboring dense populations of species clearly impoverished by fishing in unprotected environments. The six species considered in this study are high-trophic-level predators. *D. dentex* and *D. labrax* are strictly fish-eating, *E. marginatus* is a strict carnivore that includes cephalopods, other mollusks, fish and crustaceans in its diet, *S. aurata* feeds mainly on mollusks, and *D. cervinus* and *S. umbra* feed on different types of invertebrates. Given the dramatic increase of biomass, it seems evident that the fish biomass in the MR tends to move toward predators, reconstituting the trophic level of pristine areas and reversing the familiar trajectory of *fishing down the food webs* that occurs in all ecosystems exploited by fishing [56] in an example of what might be called *rebuilding up the food webs* inside MPAs. Currently, predators comprise 49% of the littoral fish community biomass in the Medes reserve [57], [39], pointing the way to a full functional recovery of the ecosystem with an inversion of the trophic pyramid [58].

This inversion could have significant flow-on consequences to the trophic interactions within the MR and has already been documented in the relationships between algae, urchins and fish [59], [60] or in the decrease of lobster recruits (*P. elephas*) [61]. Although Macpherson et al. [62] showed that there were no significant effects of increased predators on the mortality of *settlers* of bream (*Diplodus sargus*, *D. puntazo* and *D. vulgaris*) in Medes Islands MPA and other protected areas nearby, some correlational evidence suggests that the effects on the fish community in the MPAs should be notable [63]. Some authors have shown that fewer juveniles of certain species exist in the MPAs than in neighboring areas open to fishing [33], [20] and that some small benthic species, such as *Gobius bucchichi* [64] are less abundant and larger in MPAs.

### Spillover

The differences between the protected area of the Medes and the coast are large and sustained over time. It can be concluded, therefore, that after 25 y of protection and although the values of most of the MR descriptors have already approached their carrying capacity, *spillover* from the islands to the neighboring coast was not detected or was very limited in both NR and PR.

Of the six species analyzed, only three (*E. marginatus, D. dentex* and *S. umbra*) show a positive trend, which is limited to PR. Furthermore, the biomasses of *D. labrax* and *S. umbra* presented a significant interaction of protection with time, but in only two cases (*E. marginatus* and *S. umbra*) could this pattern be attributed to *spillover*. The incremental change in *D. dentex* cannot be attributed to *spillover* but to the regional increase in the species that became more pronounced in 2009; these increases have also been detected not merely in MR and PR but has also been observed in catches from the nearby port of Palamós (Gordoa, pers obs.) and other Mediterranean Spanish ports [65].

The results are similar to those obtained by Harmelin-Vivien et al. [37] in the same area when studying biomass gradients as evidence of *spillover*. These authors found that the most pronounced gradient was between MR and PR, suggesting that *spillover* was rather modest and limited to in a relatively small spatial scale within the PR.

This lack of recovery of fish populations in PR could be explained by two non-exclusive hypotheses involving the topography of the area, and fish behavior. First, the strip of sand separating rocky bottoms of MR and PR can act as a very efficient barrier for fish [66], [67]. Evidence of *spillover* has been documented only in the vicinity of marine reserves that had no important discontinuities in exploited areas [68], [69], and it is therefore not surprising that MPAs located near islands produce more acute results for rebuilding [44], [39], [70]. According to this hypothesis, a discontinuity of the substrate is not conducive to *spillover* and maximizes the role of the MPA as a refuge for species most vulnerable to fishing, while continuity of habitat favors *spillover* but dilutes the effect of protection [71], [66]. The decision to promote either spillover or rebuilding is an important management consideration based on fisheries or conservation objectives.

Secondly, it is possible that the spillover from MR to PR exists, but the continuity of the rocky substrate between PR and NR promotes migration out of the partially protected area where fish could be extracted at a rate much higher than before, so that an increase in NR cannot be detected, and a very limited increase in PR is noticed.

Regardless of the mechanism, apart from a very slight recovery of *E. marginatus*, none of the descriptors analyzed showed a positive trend in NR, and it is clear that *spillover* is never detectable in this area. This finding indicates that either there was no *spillover* from MR to NR (or from PR to NR), or if produced, the increase in abundance outside the MR due to spillover was completely offset by the effects of fishing.

Because artisanal and recreational fishing permitted in PR and NR and underwater spearfishing is authorized only in NR, the results obtained should make it possible to ascertain the relative effect of these modes of fishing on coastal fish fauna. Strictly, the lack of statistically significant differences in any of the descriptors compared between PR and NR suggest that underwater fishing may be a relatively minor impact compared with artisanal fishing. However, the dramatic effects of spearfishing on some of these species have been well established [72]. Furthermore, there is convincing evidence that *E. marginatus* can maintain high densities in partially protected areas, such as Cabrera and Port-Cros, where different types of fishing, except spearfishing, are allowed [73], [74], [46], [75].

Like many long-term studies, this work has a few important limitations. Detailed monitoring began a few years after the implementation of the MPA, and we have no systematic baselines before protection. Our results highlight that it is important to adapting the time scale of studies to the timing of natural processes and of integrating inter-annual variations, which can be pronounced in long-term trends. This is particularly true when the species involved are long-lived or slow growing. In these situations, snapshot or short-duration studies may lead to a false interpretation of ecological trends. Much of the scientific literature, including many studies assessing the effectiveness of MPAs, suffers from this downfall.

Long-term monitoring is doubtless monotonous and not well rewarded in a scientific career, where the pressure to publish appears to outweigh the need to investigate true ecological patterns. Long-term monitoring studies have been left out of science at a time when it is becoming even more evident of the temporal scales required to understand critical processes, making a historical perspective more necessary than ever. It is also very difficult for governments to accept the challenge of sustaining financing of long-term studies.

Perhaps the requirement to maintain monitoring studies is to minimize major drawbacks: such studies must be relatively inexpensive to fund, require limited effort and be easily transferable to future generations. We hope that this work will contribute to these ends.

## Supporting Information

Appendix S1 **Parameter estimates for the 4 possible models describing the relationship between different variables and the years of total protection in the MR. Unrealistic estimates of K are in brackets.**
(DOCX)
