# Supplementary material for: Long-Term Recovery Patterns and Limited Spillover of Large Predatory Fish in a Mediterranean MPA
Source: PLoS One. 2013 Sep 12;8(9):e73922. doi: 10.1371/journal.pone.0073922 (PMC3771876; doi:10.1371/journal.pone.0073922)
Supplement: Appendix S1 — Parameter estimates for the 4 possible models describing the relationship between different variables and the years of total protection in the MR. Unrealistic estimates of K are in brackets. (DOCX) [file pone.0073922.s001.docx]

|  | **Linear** |  |  | **Exponential** |  |  | **V. Bertalanffy** |  |  |  | **B.f. Logistic** |  |  |  |
| --- | --- | --- | --- | --- | --- | --- | --- | --- | --- | --- | --- | --- | --- | --- |
|  | **a** | **b** | **R^2^** | **a** | **r** | **R^2^** | **K** | **r** | **t_0_** | **R^2^** | **K** | **y_0_** | **r** | **R^2^** |
| **Species richness** | 2.52 *** | 0.005 | 0.03 | 2.53 *** | 0.002 | 0.03 | 2.65*** | 0.50 | -4,71 | **0.12** | 2.65 *** | 0.11 | 0.62 | 0.12 |
| **Total abundance** | 3.92 * | 0.30 * | 0.47 | 5.36 *** | 0.030 ** | 0.43 | 10.94 *** | 0.71 | -6.16 * | **0.58** | 10.83 *** | 0.72 | 0.28 | 0.57 |
| **Total biomass** | 27.69 ** | 1.14 ** | 0.37 | 31.9 *** | 0.023 * | 0.35 | 59.09 ** | 0.11 | -0.79 | 0.41 | 57.26 *** | 14.77 | 0.16 | **0.42** |
| ***E. marginatus* (ab)** | 0.86 * | 0.09 *** | 0.61 | 1.33 *** | 0.035 *** | 0.56 | 3.25*** | 0.13 | -4.41 | 0.68 | 3.13 *** | 0.34 | 0.22 | **0.68** |
| ***D. dentex* (ab)** | -0.30 | 0.06 * | 0.61 | 1.18 *** | 0.080 *** | **0.61** | 15.18 | 0.004 | -5.16 | 0.60 | (39089.0) | 0.18 | 0.08 | (0.61) |
| ***D. cervinus* (ab)** | 1.05 * | 0.01 | 0.03 | 1.09 *** | 0.008 | 0.03 | 1.32 | 0.77 | -8.11 *** | **0.28** | 1.31 *** | 0.0004 | 1.15 | **0.28** |
| ***D. labrax* (ab)** | 0.52 | 0.07 | 0.11 | 1.00 | 0.032 | 0.09 | 2.11 *** | 0.38 | -8.78 *** | 0.23 | 2.08 *** | 0.0003 | 0.79 | **0.24** |
| ***S. umbra* (ab)** | 0.30 | 0.11 *** | 0.62 | 0.91 *** | 0.048 *** | 0.61 | 7.03 | 0.02 | -0.39 | **0.63** | 3.96 | 0.57 | 0.11 | 0.62 |
| ***S. aurata* (ab)** | 1.35 *** | -0.04 * | 0.38 | 1.86 ** | -0.057 | **0.43** | 86.44 | 3.03 | 89.49 | 0.00 | 0.18 | 196.6 | 0.02 | **0.46** |
| ***E. marginatus* (bio)** | 19.85 * | 0.76 * | 0.27 | 22.7 *** | 0.021 * | 0.25 | 37.07 *** | 0.24 | -4.70 | 0.38 | 37,28 *** | 5.19 | 0.27 | **0.38** |
| ***D. dentex* (bio)** | -2.66 * | 0.33 *** | 0.73 | 0.35 * | 0.114 *** | **0.78** | 182.60 * | 0.002 | -8.16 | 0.73 | (332274.1) | 0.40 | 0.10 | (0.78) |
| ***D. cervinus* (bio)** | 2.27** | 0.04 | 0.05 | 2.34 ** | 0.011 | 0.04 | 3.08 *** | 0.58 | -7.71 | 0.22 | 3.07 *** | 0.002 | 0.83 | **0.28** |
| ***D. labrax* (bio)** | 0.33 | 0.13 | 0.20 | 1.29 | 0.040 | 0.17 | 3.51 ** | 0.19 | -7.77 * | 0.27 | 3.40 *** | 0,04 | 0.38 | **0.28** |
| ***S. umbra* (bio)** | 1.03 | 0.15 | 0.46 | 1.79 ** | 0.034 ** | 0.44 | 5.56 | 0.08 | -3.06 | 0.46 | 5.16 * | 0,85 | 0.15 | **0.47** |
| ***S. aurata* (bio)** | 2.51 *** | -0.05 | 0.16 | 2.95 ** | -0.035 | **0.18** | 1.60 | - | - | - | 1.05 | 331.9 | 0.07 | (0.20) |
